# Photoinduced effects of m-tetrahydroxyphenylchlorin loaded lipid nanoemulsions on multicellular tumor spheroids

**DOI:** 10.1186/s12951-016-0221-x

**Published:** 2016-09-07

**Authors:** Doris Hinger, Fabrice Navarro, Andres Käch, Jean-Sébastien Thomann, Frédérique Mittler, Anne-Claude Couffin, Caroline Maake

**Affiliations:** 1 Institute of Anatomy, University of Zurich, Winterthurerstrasse 190, 8057 Zurich, Switzerland; 2 Technologies for Biology and Healthcare Division, CEA, LETI, MINATEC Campus, Commissariat à l’Énergie Atomique et aux Énergies Alternatives (CEA), 38054 Grenoble, France; 3 Université Grenoble Alpes, 38000 Grenoble, France; 4 Center for Microscopy and Image Analysis, University of Zurich, Winterthurerstrasse 190, 8057 Zurich, Switzerland

**Keywords:** Nanoemulsion, Biocompatibility, mTHPC, Photodynamic therapy, Spheroids, Lipid nanoparticles

## Abstract

**Background:**

Photosensitizers are used in photodynamic therapy (PDT) to destruct tumor cells, however, their limited solubility and specificity hampers routine use, which may be overcome by encapsulation. Several promising novel nanoparticulate drug carriers including liposomes, polymeric nanoparticles, metallic nanoparticles and lipid nanocomposites have been developed. However, many of them contain components that would not meet safety standards of regulatory bodies and due to difficulties of the manufacturing processes, reproducibility and scale up procedures these drugs may eventually not reach the clinics. Recently, we have designed a novel lipid nanostructured carrier, namely Lipidots, consisting of nontoxic and FDA approved ingredients as promising vehicle for the approved photosensitizer m-tetrahydroxyphenylchlorin (mTHPC).

**Results:**

In this study we tested Lipidots of two different sizes (50 and 120 nm) and assessed their photodynamic potential in 3-dimensional multicellular cancer spheroids. Microscopically, the intracellular accumulation kinetics of mTHPC were retarded after encapsulation. However, after activation mTHPC entrapped into 50 nm particles destroyed cancer spheroids as efficiently as the free drug. Cell death and gene expression studies provide evidence that encapsulation may lead to different cell killing modes in PDT.

**Conclusions:**

Since ATP viability assays showed that the carriers were nontoxic and that encapsulation reduced dark toxicity of mTHPC we conclude that our 50 nm photosensitizer carriers may be beneficial for clinical PDT applications.

**Electronic supplementary material:**

The online version of this article (doi:10.1186/s12951-016-0221-x) contains supplementary material, which is available to authorized users.

## Background

A wealth of publications report on the development of promising novel nanoparticulate drug carriers including liposomes [1], polymeric nanoparticles [2], metallic nanoparticles [3] and lipid nanocomposites [4]. However, many of them contain components that would not meet safety standards of regulatory bodies such as the European Medicines Agency (EMA) or the US food and drug administration (FDA) [5]. Furthermore, due to difficulties of the manufacturing processes, reproducibility and scale up procedures these drugs may eventually not translate into the clinics.

Recently, we have designed a novel lipid nanostructured carrier, namely Lipidots, consisting of nontoxic and FDA approved ingredients: wax and soybean oil serve as core components and lecithin as membranous hull with a polyethylene glycol (PEG) coating [6]. Containing only natural compounds, they are likely to be broken down and removed or recycled by the body [7]. Lipidots may be utilized and adapted for many different applications such as fluorescent imaging probes, contrast agent carriers, or targeted drug delivery [8]. They offer the possibility to tune the viscosity of their lipid core, thereby adapting the release of an encapsulated compound to the desired profile [9]. Moreover, Lipidots can be manufactured with high colloidal stability at laboratory and industrial scales using ultrasonics or high pressure homogenization [6].

An interesting future application of Lipidots may be in the context of photodynamic therapy (PDT), a modality which is currently receiving increasing clinical attention as a promising anti-cancer treatment [10]. PDT principles rely on the activation of a light-sensitive drug (the photosensitizer, PS), which, through oxidative reaction cascades of type I and type II leads to the generation of cytotoxic reactive oxygen species (ROS) and strictly localized cell death. Remarkably, PDT has the potential to overcome disadvantages of standard oncologic regimes such as surgery, chemo- or radiotherapy because it is minimal invasive, bears little risk for the development of resistance and lacks severe side effects [11]. However, the efficiency of PDT critically depends on a high local accumulation of the PS at the tumor site. But since many potent PSs are hydrophobic, they tend to aggregate in aqueous environments (e.g. after intravenous injection), with negative consequences for their biodistribution and photoactivity, which can eventually lead to unsatisfactory therapeutic effects [12]. With the aim to improve PDT applications, various PSs have been entrapped into nanocarriers, including e.g. Photophrin, hypocrellin A, chlorin e6, tetraarylporphyrin, the near infrared dye indocyanine green [13] or the powerful FDA approved second generation PS m-tetrahydroxyphenylchlorin (mTHPC) [14].

In a previous study we have reported on the successful and reproducible encapsulation of mTHPC (generic name: Temoporfin) into Lipidots and their extensive characterization [15]. While our physico-chemical and photophysical data indicate that these particles may be well suited for PDT applications, results about their biological activity are only very preliminary yet [15]. In the present study we have thus set out to investigate PDT effects of mTHPC-loaded Lipidots for the first time in an advanced in vitro 3-dimensional (3D) head and neck cancer cell model. To estimate their potential for clinical PDT use, we produced Lipidots with two sizes (50 and 120 nm) and, after mTHPC encapsulation, compared their in vitro effects to free mTHPC in terms of light-induced toxicity, penetration properties, dispersion behaviour, PDT effects, cell death mechanisms and gene expression patterns.

## Methods

### Chemicals

MTHPC was obtained from Biolitec, Jena, Germany as powder. A stock solution of 1.47 mM (1 mg/mL) in 100 % ethanol was prepared and stored at 4 °C in the dark. 1,1′-dioctadecyl-3,3,3′,3′-tetramethylindodicarbocyanine perchlorate (DiD) was purchased from Life Technologies (Carlsbad, USA). If not otherwise indicated, chemicals were purchased from Sigma-Aldrich, Buchs, Switzerland.

### Nanoparticle preparation

Lipidots were prepared according to Delmas et al. [9] and Navarro et al. [15]. Briefly, the manufacturing process consists of mixing an aqueous phase and a lipid phase which are separately prepared, including on the one hand MyrjS40 surfactant dissolved into 1X phosphate buffered saline (PBS) (154 mM NaCl, 0.1 M Na_2_HPO_4_, pH 7.4) and on the other hand soybean oil and wax (Suppocire NB) under melted state. The ultrasonication step is performed using a VCX750 ultrasonic processor during 20 min (power input 190 W, 3-mm probe diameter, Sonics). MTHPC was incorporated into the lipid mixture as a concentrated solution in ethyl acetate and after vacuum elimination of organic solvent, the oily phase was added to the aqueous phase and emulsification was performed as previously described [15]. For 50 nm Lipidots, the dispersion is composed of 37.5 % (w/w) of lipid phase (with a lecithin/PEG surfactant weight ratio of 0.19 and a surfactant/core weight ratio of 1.20) whereas for 120 nm Lipidots, the dispersion is composed of 43.0 % (w/w) of lipid phase (with a lecithin/PEG surfactant weight ratio of 0.21 and a surfactant/core weight ratio of 3.0). The Lipidots were loaded with mTHPC (thereafter called M-Lipidots) at two different ratios of numbers of PS per nanoparticle for 50 and 120 nm-sized Lipidots, respectively (920 and 4600 molecules of mTHPC/particle, respectively). The mTHPC concentrations were determined by high-performance liquid chromatography (HPLC) analysis. HPLC of prepared samples was carried out on a Sunfire C18 column (250 mm × 4.6 mm, i.d. 5 µm) at 30 °C. The mTHPC compound was eluted at 2.10 min using a isocratic mobile phase of acetonitrile/H_2_O trifluoroaceticacid, 0.1 %: 9/1 at 1 mL/min flow rate after injection of 30 µL. The UV detection is operated at 425 nm. The mTHPC concentrations were assessed using a calibration curve in the range of 1–12 µg/mL. For comparisons at constant PS content, all working solutions were diluted using PBS to obtain equivalent mTHPC amounts in solution to be added in cell culture media for PDT treatment (3.67, 7.34, 14.69 µM mTHPC content). For in vitro additional fluorescence imaging and flow cytometry purposes, dye-doped nanoparticles, thereafter called D-Lipidots, were prepared as previously described [16] by incorporating DiD lipophilic indocyanine into the oily core of 50 nm Lipidots.

### Monolayer cell culture

CAL-33 tongue squamous cell carcinoma cells (DSMZ, Braunschweig, Germany), were grown in RPMI without phenol red, 10 % FCS, 2 mM Glutamax (Life Technologies), and 1 % Penicillin/Streptomycin (LifeTechnologies). Cells were kept in 75 cm^2^ cell culture flasks at 5 % CO_2_ and 37 °C. Cell counting was performed with a Neubauer chamber (Laboroptik Ltd., Lancing, UK) on an aliquot of cells after staining with 0.1 % (w/v) nigrosin in PBS.

### Spheroid cell culture

The bottoms of 96 well plates were coated with 65 µL 1.5 % (w/v) agarose (Life Technologies) in cell culture medium without supplements. 3D cell culture spheroids were prepared by putting 96 drops of 5000 CAL-33 cells in 10 µL complete cell culture medium on the inner side of the lid of a 96 well plate. Then the lids with the hanging drops were put back on the plates and incubated for 24 h. Thereafter, 190 µL of complete cell culture medium was added to the wells and the drops were spun down shortly in a centrifuge (Virion, Zürich, Switzerland) and incubated for another 72 h. By that time the spheroids had reached an average diameter of 200 µm and were immediately used for the experiments [17].

### Light microscopy

#### Monolayer cells

CAL-33 cells were seeded on 12 mm glass cover slips (Karl Hecht, Sondheim, Germany) and incubated with 7.34 µM mTHPC or M-Lipidots or 1 µM D-Lipidots in cell culture medium for up to 28 h in the dark. The cover slips were washed twice with PBS and subsequently fixed for 20 min with 4 % (w/v) formaldehyde (FA)/PBS. After washing they were mounted on microscopic slides (Menzel, Braunschweig, Germany) with Glycergel (Dako, Glostrup, Denmark) and analyzed with a confocal laser scanning microscope (Leica SP5, Heerbrugg, Switzerland). MTHPC was excited at 488 nm and fluorescence was detected between 590–660 nm. Images were analyzed with the imaging software Imaris (Bitplane, Belfast, UK).

#### Spheroids

Spheroids were incubated with 7.34 µM of mTHPC or M-Lipidots in 100 µL cell culture medium for up to 28 h in 96 well plates in the dark. Spheroids were picked with a 1 mL pipette and transferred to microcentrifuge tubes. After washing twice with PBS spheroids were fixed in 4 % (w/v) FA/PBS for 1 h, washed in PBS and analyzed in 18-well µ-slides (IBIDI) by widefield fluorescence microscopy (Leica DMI 6000) or confocal laser scanning microscopy (Leica, SP5). Per time point, 3–5 images were acquired using differential interference contrast (DIC) and epifluorescence and mean fluorescence was calculated from regions of interest (ROIs) which were drawn around the cell assemblies in the DIC channel with Leica AS lite software. Confocal laser scanning microscopy (Leica SP5) was performed on 3–5 fixed spheroids per condition with a 20× objective (HC Plan APO). After spheroid integrity was confirmed by DIC imaging, optical sectioning was performed with an argon laser at 488 nm for excitation of mTHPC. Pictures from the center of the spheroids were taken and processed with the imaging software Imaris (Bitplane, Belfast, UK).

### Cytotoxicity assessment

Spheroids were incubated with 3.67, 7.34 and 14.69 µM of mTHPC or M-Lipidots for 24 h in 96 well plates in the dark. Substance-mediated damage (i.e. dark toxicity) was assessed by either measuring spheroid areas as ROIs with widefield microscopy and the Leica AS imaging software or by means of an ATP luciferase viability assay (Promega, Fitchburg, USA). For the ATP luciferase viability assay 100 µL of Cell Viability Assay solution was added to each well after drug incubation, the contents were mixed by pipetting and the plate was transferred for 20 min to a shaker. Subsequently bioluminescence was measured in a microplate reader (Biotek, Vermont, USA).

### Phototoxicity assessment

Spheroids were incubated with 3.67, 7.34 and 14.69 µM of mTHPC or M-Lipidots for 24 h in 96 well plates in the dark. Subsequently the plates were subjected to PDT by illuminating with white light from 2.5 cm above (3440 lx; fluorescent tube SYLVA-NIA standard F15 W/154, daylight) for 20 min. To ensure an even illumination, the outer rim of the well plates was never used for experimentation and the sequence of samples within the plate was changed between repetitions. Spheroid areas were microscopically determined as described above and cell survival was determined by ATP luciferase viability assay 5 h after irradiation as described above.

### Apoptosis assay

Spheroids were incubated with 7.34 µM mTHPC or M-Lipidots for 24 h. After illuminating for 1 min (conditions as described above) spheroids were incubated for another 1.5 h with 100 µL 15 µM Hoechst 33342 and 30× Flica reagent (FAM Flica Poly Caspase kit, ImmunoChemistry Technologies, Enzo Life Sciences, Lausen, Switzerland). The spheroids were subsequently harvested with a 1 mL pipette and transferred to microcentrifuge tubes. After washing twice with wash buffer (FAM Flica Poly Caspase Kit) they were fixed for 1 h in fixing solution (FAM Flica Poly Caspase Kit) and analyzed in 18 well µ-slides (IBIDI) with a confocal laser scanning microscope (Leica SP5, Heerbrugg, Switzerland) within 24 h.

### Electron microscopy

Spheroids were incubated for 24 h with 3.67 µM mTHPC or 50 nm M-Lipidots and irradiated for 1 min as described above. One hour after light treatment they were washed and fixed and sequentially treated with OsO_4_ and uranylacetate. After dehydration they were embedded in Epon/Araldite and sections were contrasted with uranyl acetate and lead citrate. They were examined with a CM100 transmission electron microscope (FEI, Eindhoven, The Netherlands) or with an Auriga 40 scanning electron microscope (Zeiss, Oberkochen, Germany). For a more detailed description see Additional file 1.

### Quantitative reverse transcription polymerase chain reaction (qRT-PCR)

A total of 120 spheroids were incubated with 3.67 µM mTHPC or 50 nm M-Lipidots for 24 h. After illuminating for 1 min as described spheroids were incubated for another 2 h, subsequently harvested with a 1 mL pipette and transferred to microcentrifuge tubes. They were washed twice with PBS and resuspended in 600 µL lysis buffer (Qiagen, Venlo, The Netherlands), vortexed vigorously and passed 30 times through a 1 mL syringe with a 20 gauge needle. Total RNA was extracted with the RNeasy Micro Kit (Qiagen) as described per manufacturer’s instructions, processed with a cNDA synthesis kit (Qiagen) and the obtained cDNA used for a quantitative PCR array (Human Cancer Drug Targets RT^2^ Profiler PCR Array, Qiagen). For further details please refer to the Additional file 1.

### Flow cytometry

Flow cytometry analysis of the interaction of fluorescent D-Lipidots with cells was performed using a 9 colors FACS BD LSR2 equipped with lasers emitting at 488 and 633 nm (BD, Franklin Lakes, USA). CAL-33 cells were seeded at a density of 10^5^ cells per well in 12 well plates and incubated for 24 h. D-Lipidots with a diameter of 50 nm were incubated at the corresponding concentration of 1 µM DiD in presence of cell monolayers for 2, 3 or 6 h in complete cell culture medium. Thereafter, cells were rinsed with PBS (×2), harvested by the addition of trypsin followed by a centrifugation, and then fixed with 2 % FA before flow cytometry analysis. 10,000 to 20,000 events were recorded. The data from fluorescence measurements at an emission wavelength of 660 nm for DiD were analyzed using DIVA v8.1 software (BD) by using the overlay option.

### Statistical evaluation and graphical modelling

Two-way ANOVA of cell toxicity and phototoxicity data was analyzed from at least two independent experiments and five replicates per condition. Means are plotted ± standard deviations. Statistics and graphical plots were established and analyzed with GraphPad Prism software (Graphpad Software, La Jolla, USA).

## Results

### Nanoparticle preparation

To investigate the effect of particle size and PS payload on transport and delivery, two series of nanoparticles were prepared with two different payloads. For the 50 nm nanoparticles, mTHPC was incorporated with a content of 920 molecules/particle whereas for 120 nm particles, the amount of mTHPC was estimated at 4600 molecules/particle. Therefore one 120 nm nanoparticle contains fivefold more molecules of mTHPC than one 50 nm nanoparticle. Expressed in equivalent mTHPC concentration (3.67, 7.34 and 14.69 µM) the solution of 50 nm nanoparticles contains fivefold more particles than the solution of 120 nm particles. As observed in our preliminary study [15], mTHPC was efficiently encapsulated into lipid nanoparticles without affecting neither the colloidal properties of the carrier nor photophysical properties of the loaded PS. Indeed, an aggregation of mTHPC inside the lipid core of nanoparticles can be observed only for 50 nm particle at high payload (>4 %w/w total lipid, data not published). Estimated from the whole excipients initially incorporated in the Lipidot formulation, mTHPC was loaded in our study at 2.8 and 1.0 % w/w for 50 and 120 nm particles, respectively (Table 1).

**Table 1:** Physicochemical characterization of Lipidots

	Lipid (mg/mL)	Numberof particles/mL	MTHPC molecules/particle	mTHPC (µg/mL)	Drug loading^a^	Hydrodynamic diameter (nm)	Poly-dispersity index
50 nm M-Lipidot	50	7.27565 × 10^14^	~920	722	2.8 %	47.7 ± 1.1	0.153 ± 0.01
120 nm M-Lipidot	50	5.26306 × 10^13^	~4600	262	1.0 %	111.2 ± 2.2	0.103 ± 0.01
50 nm Lipidot^b^	50	7.27565 × 10^14^	–	–	–	49.5 ± 1.5	0.170 ± 0.07
120 nm Lipidot^b^	50	5.26306 × 10^13^	–	–	–	95.4 ± 3.4	0.120 ± 0.05

Data with standard deviation

^a^Expressed w/w of total lipids (included in nanoparticle formulation)

^b^Empty Lipidots

### Particle size and size distribution of lipid nanoparticles

Dynamic light scattering (DLS) technique was used to determine the particle hydrodynamic diameter (in nm), particle size distribution (expressed by polydispersity index PDI) using Zetasizer Nano ZS (Malvern Instruments, France). At least three different nanoparticle batches (lipid dispersed phase weight fraction: 10 %) are measured per condition. Data were expressed as mean ± standard deviation of three independent measurements performed at 25 °C (Table 1).

### Lipidot size drives uptake kinetics in CAL-33 cells

**Fig. 1:**
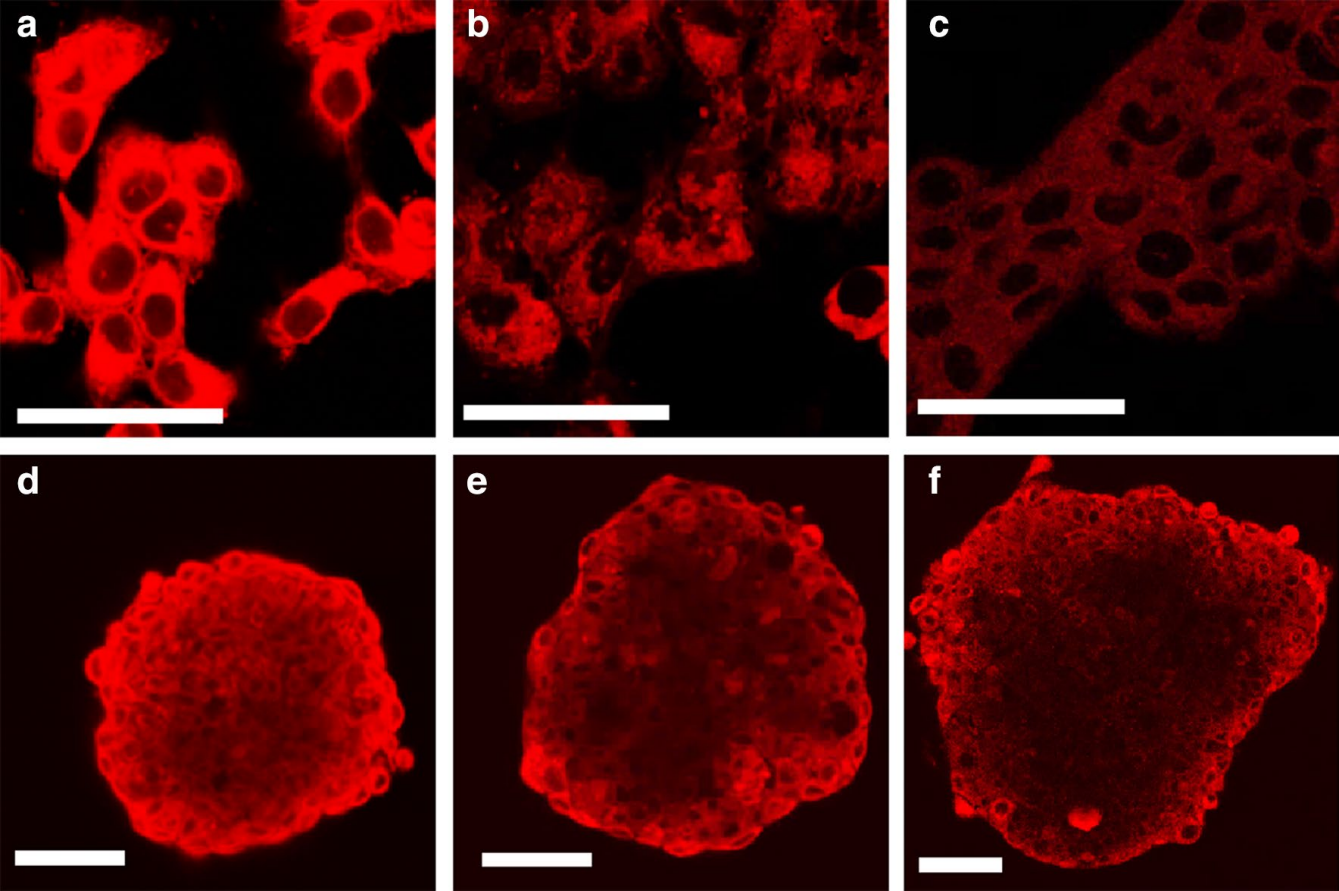
Confocal laser scanning microscopy images of CAL-33 cells incubated for 28 h with free mTHPC (**a**, **d**), 50 nm M-Lipidots (**b**, **e**) and 120 nm M-Lipidots (**c**, **f**) in monolayers (**a**–**c**) and spheroids (**d**–**f**). Concentration for all treatments: 7.34 µM mTHPC. *Scale bar* 50 µm

Using confocal laser scanning microscopy and CAL-33 monolayers and spheroids, uptake of 50 and 120 nm M-Lipidots was investigated over time and compared to free mTHPC (Fig. 1). In CAL-33 monolayer cultures, fluorescence of free mTHPC could be readily detected after 2 h of incubation as a diffuse signal throughout the cytoplasm, sparing the nucleus. In contrast, no fluorescence from our nanoparticle formulations was apparent at this time point. Only after 6 h both sizes of M-Lipidots were visible with the same distribution pattern as free mTHPC, however, the fluorescence was markedly weaker with 120 nm M-Lipidots compared to 50 nm M-Lipidots. The intracellular distribution pattern stayed similar until 28 h but fluorescence accumulated over time for all formulations (Fig. 1a–c).

**Fig. 2:**
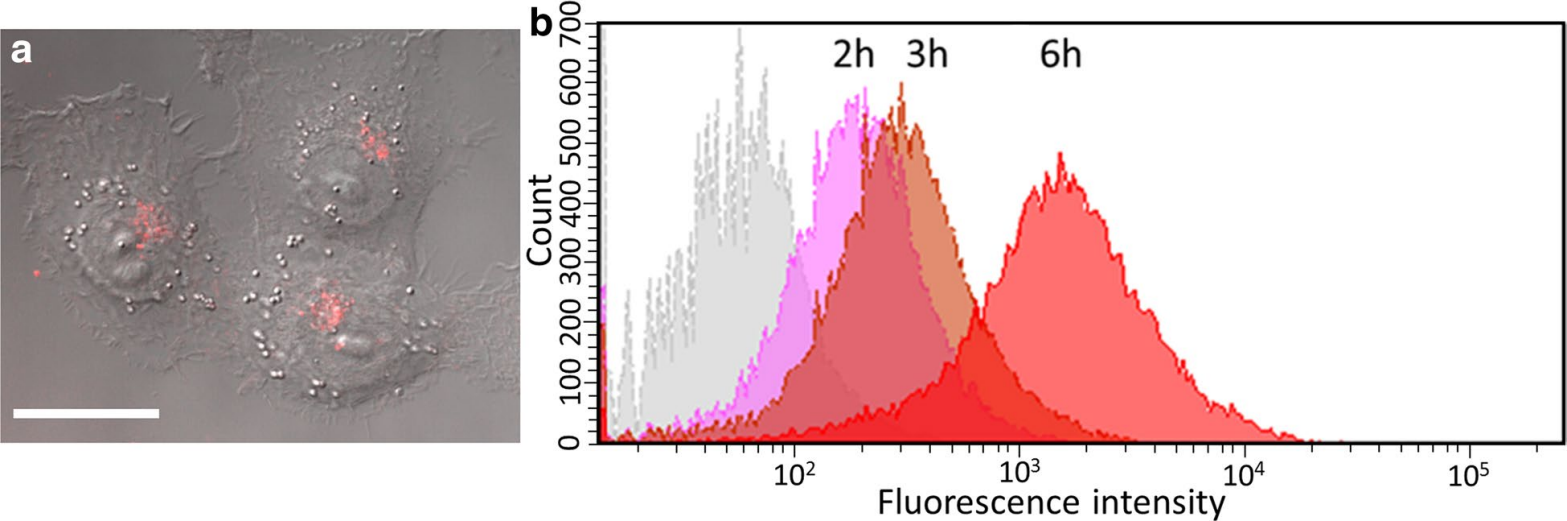
**a** Confocal laser scanning microscopy image of CAL-33 cells incubated with 1 µM D-Lipidots (50 nm) for 6 h. *Scale bar* 20 µm. **b** Flow cytometry analyses of CAL-33 cells incubated with 1 µM D-Lipidots (50 nm) for 2 h (*pink*), 3 h (*light brown*), 6 h (*red*), as compared to control (*grey*)

To obtain further information with regard to uptake kinetics, flow cytometry was used to measure in CAL-33 the fluorescence of 50 nm D-Lipidots over time (Fig. 2). These 50 nm D-Lipidots show the same accumulation behavior as 50 nm M-Lipidots (Fig. 2a), but are better suited for flow cytometry applications. Data confirmed microscopic observations in CAL-33 cells, showing an increase of fluorescence intensity after 6 h of incubation as compared to earlier time points (Fig. 2b).

To better predict the in vivo behavior, uptake was then investigated in CAL-33 spheroids (Fig. 1d–f). In this 3D model of an avascular mini tumor, free mTHPC accumulated in the outer cell layer at about the same time as in monolayer cells (2 h), however, it took up to 6 h until the PS was penetrating further into the spheroid. Eventually it reached the spheroid core at 24 h with a modest overall fluorescence increase until 28 h. At these late time points, fluorescence signals showed a homogeneous distribution within the spheroid. The weaker fluorescent signals of 50 nm M-Lipidots were apparent in the outer cell layers after 4 h, and continued to penetrate slowly deeper into the spheroid center. At 28 h the core was fluorescent, but the signal displayed a more punctuate and less homogeneous pattern. Compared to 50 nm M-Lipidots, penetration of 120 nm M-Lipidots was retarded, most of which did not reach the center even at 28 h as evidenced by a less fluorescent spheroid core.

**Fig. 3:**
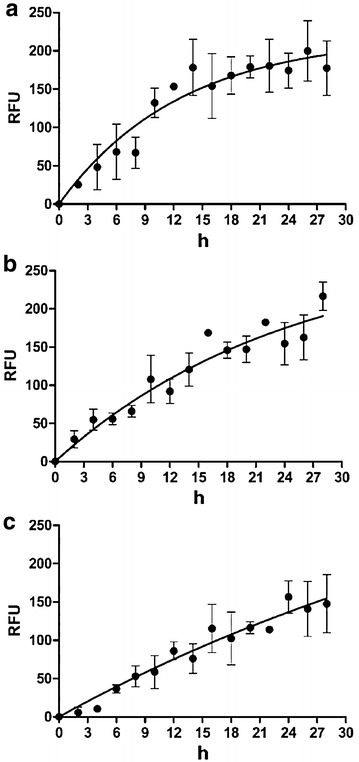
Time dependent uptake curves of free mTHPC (**a**), 50 nm M-Lipidots (**b**) and 120 nm M-Lipidots (**c**) established by widefield fluorescence measurement in CAL-33 spheroids. *RFU* relative fluorescence units. Concentration for all treatments: 7.34 µM mTHPC

Semiquantitative analyses of microscopy data confirmed that time dependent uptake curves were different between free mTHPC and M-Lipidots in the spheroid model (Fig. 3). Free mTHPC was taken up in a nonlinear, asymptotical way with high initial uptake rates and quickly decreasing rates over time whereas 120 nm M-Lipidots were taken up by the spheroid in an almost linear fashion during the whole time of the experiment at a very low initial uptake rate. The uptake curve of the 50 nm M-Lipidots presents an uptake in a nonlinear way but at a lower initial uptake rate as free mTHPC. Based on uptake studies, further studies were therefore performed after a 24 h exposure to the compounds.

### The nanoformulations are less cytotoxic than the free substance at high drug concentrations

**Fig. 4:**
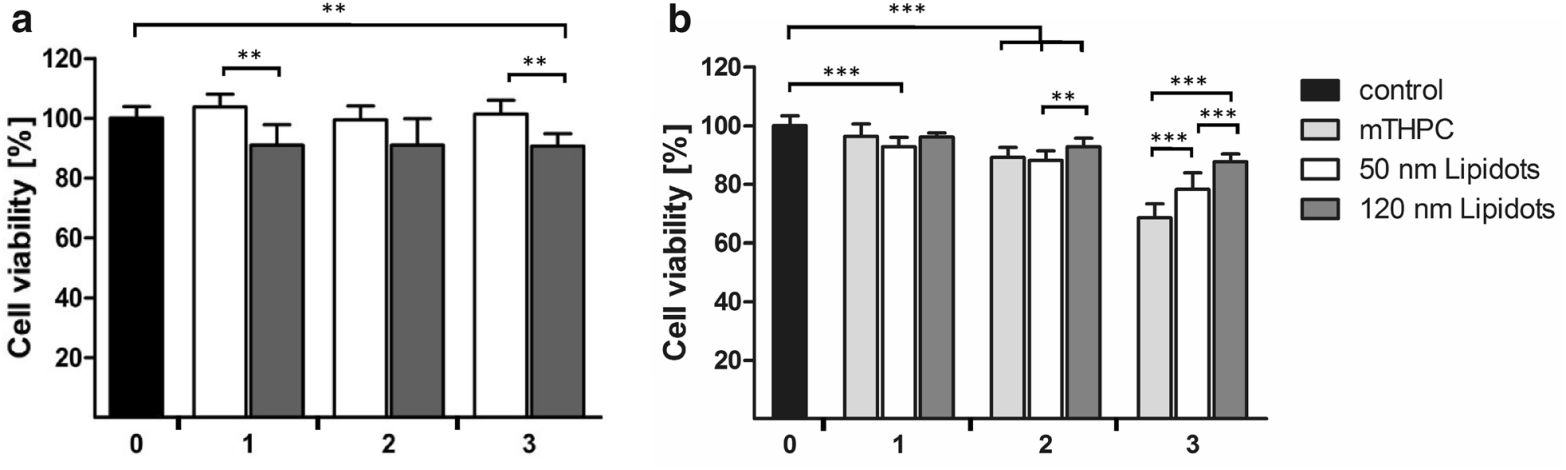
Cell viability ATP assays of CAL-33 spheroids after 24 h incubation. **a** Cytotoxic effects (dark toxicity) of empty Lipidots with an equalized amount of lipid content as in **b**. **b** Cytotoxic effects (dark toxicity) of 3.67 µM (*1*), 7.34 µM (*2*) and 14.69 µM (*3*) mTHPC or 50/120 nm M-Lipidots. **p < 0.01. ***p < 0.001

To obtain information about a possible cytotoxicity of our nanocarriers, we first tested empty Lipidots by means of an ATP luciferase viability assay that measures cell viability in CAL-33 spheroids (Fig. 4a). A comparison revealed that both 50 and 120 nm Lipidots are well tolerated for concentrations of particles corresponding to the equivalent mTHPC concentration from 0 to 14.69 µM (≙69.3–692.9 µg/mL lipid [50 nm]; 190.7 µg/mL–1.90 mg/mL lipid [120 nm]), with the smaller particles being slightly superior (p < 0.01). While the 50 nm particles did not exhibit any toxicity at the tested concentrations the 120 nm particles reduced viability by 10 %. As a next step, cytotoxic effects of PS-loaded M-Lipidots were compared to free mTHPC in CAL-33 spheroids (Fig. 4b). While free mTHPC showed a clear toxicity (68 % viability) in the dark at the highest concentration tested (14.69 µM), encapsulation of mTHPC into Lipidots resulted in a significantly reduced dark toxic effect (78 % viability with the 50 nm Lipidots; 86 % viability with the 120 nm Lipidots, p < 0.001).

### The 50 nm M-Lipidots show high photodynamic potency similar to free mTHPC

**Fig. 5:**
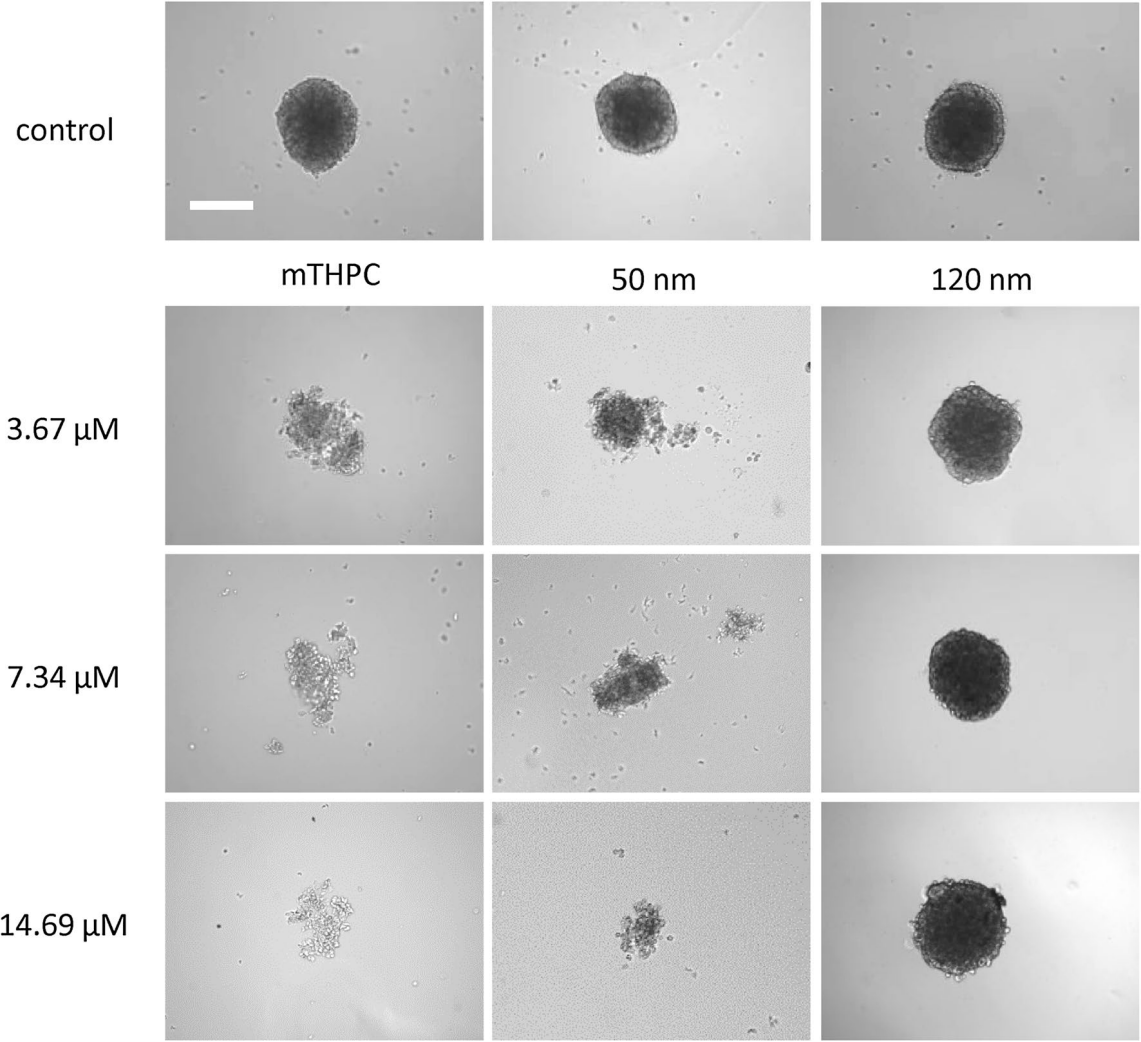
Light microscopy of CAL-33 spheroids incubated for 24 h with 3.67, 7.34 and 14.69 µM mTHPC or 50/120 nm M-Lipidots after light irradiation with 3440 lx for 20 min

**Fig. 6:**
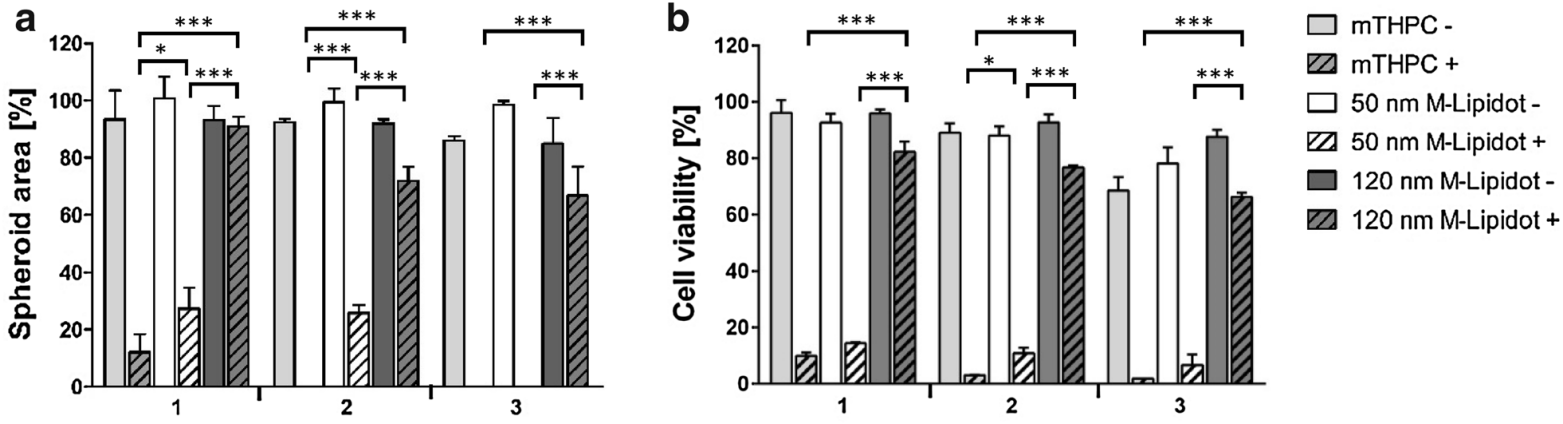
**a** Light microscopic measurements of spheroid areas of CAL-33 spheroids incubated with 3.67 µM (*1*), 7.34 µM (*2*) and 14.69 µM (*3*) mTHPC or 50/120 nm M-Lipidots with (+) and without (−) light irradiation with 3440 lx for 20 min. **b** Cell viability ATP assays of CAL-33 spheroids incubated at the same conditions as in **a**. *p < 0.05. ***p < 0.001

The PDT effects mediated by M-Lipidots or free mTHPC were investigated in CAL-33 spheroids (Figs. 5, 6). Our microscopic analyses showed that PDT with both free mTHPC and 50 nm M-Lipidots induced a pronounced and comparable destruction of the spheroids (Fig. 5). Although the size reduction was difficult to microscopically measure under conditions of high destruction, the results correlated with the respective ATP luciferase viability assays (Fig. 6b). The 50 nm Lipidots as well as free mTHPC reduced spheroid sizes by 100 % at higher concentrations (p < 0.001). However, after PDT with 120 nm M-Lipidots, even at the highest concentration (14.69 µM), only mild phototoxic effects were visible with size reductions by only 34 % (Figs. 5, 6a, p < 0.001). These limited PDT effects of 120 nm M-Lipidots could also be confirmed by ATP luciferase viability assays (Fig. 6b). Viability after PDT with the highest concentration (14.69 µM) was 1.8 % with mTHPC, 6.6 % with the 50 nm particles and 66.2 % with the 120 nm particles (p < 0.001).

### Free mTHPC causes apoptosis and necrosis while 50 nm M-Lipidots cause mostly apoptosis

**Fig. 7:**
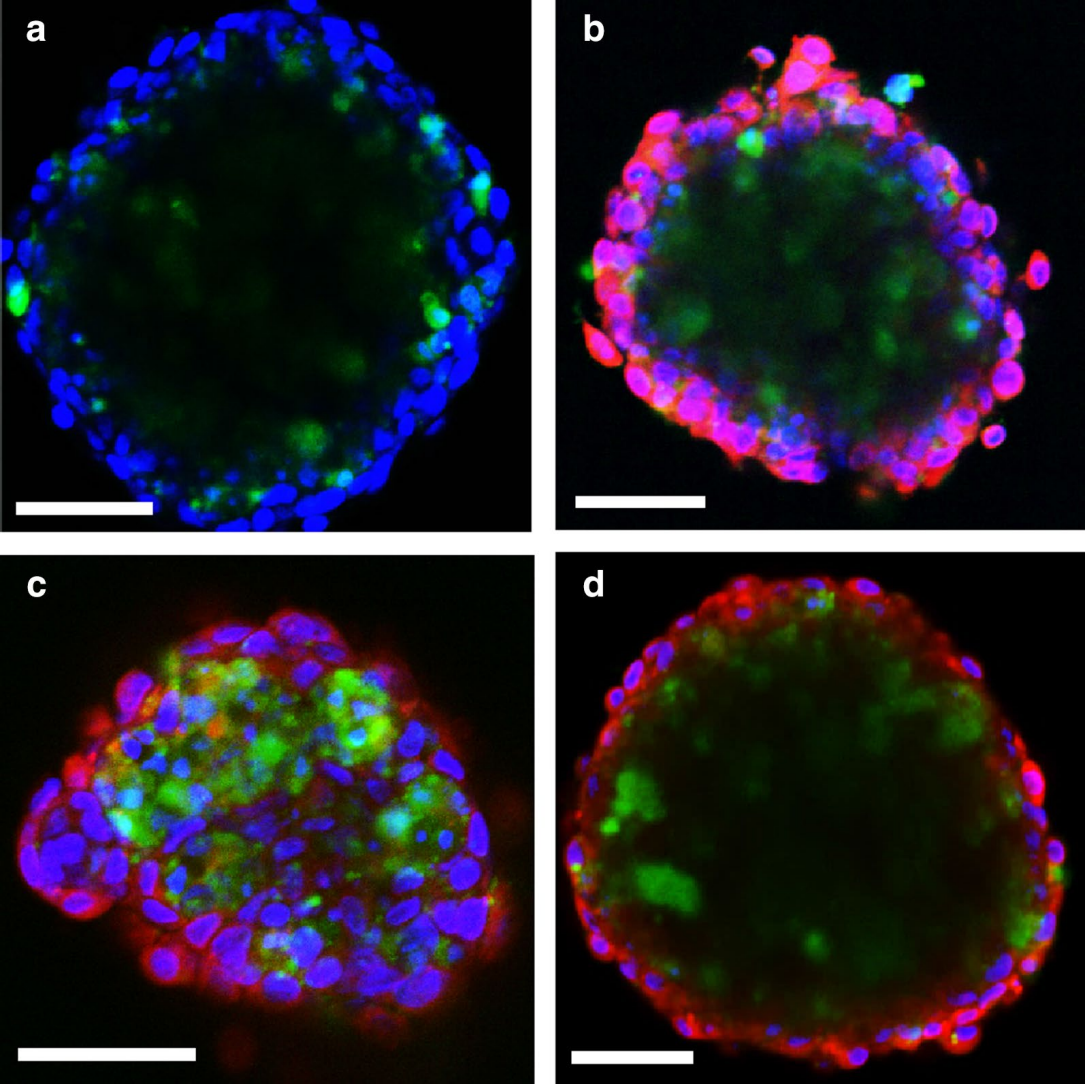
Confocal laser scanning microscopy images of the fluorescent labeled inhibitor of caspases (FLICA) apoptosis assay after irradiation of CAL-33 spheroids with 3440 lx for 1 min. FLICA: *green*, Hoechst 33342 nuclear stain: *blue*, mTHPC (*red*). Untreated control (**a**) and incubations with mTHPC (**b**), 50 nm M-Lipidots (**c**) or 120 nm M-Lipidots (**d**). Concentration for all treatments: 3.67 µM mTHPC. Incubation time 24 h. *Scale bar* 50 µm

By “FLICA” apoptosis assays high pan-caspase activity was detected in CAL-33 spheroids after PDT with 50 nm M-Lipidots (Fig. 7c) and, to a lesser extent, after treatment with free mTHPC and irradiation (Fig. 7b). Very low caspase staining occurred after PDT with 120 nm M-Lipidots (Fig. 7d) which was barely more intense than staining of control spheroids (Fig. 7a).

**Fig. 8:**
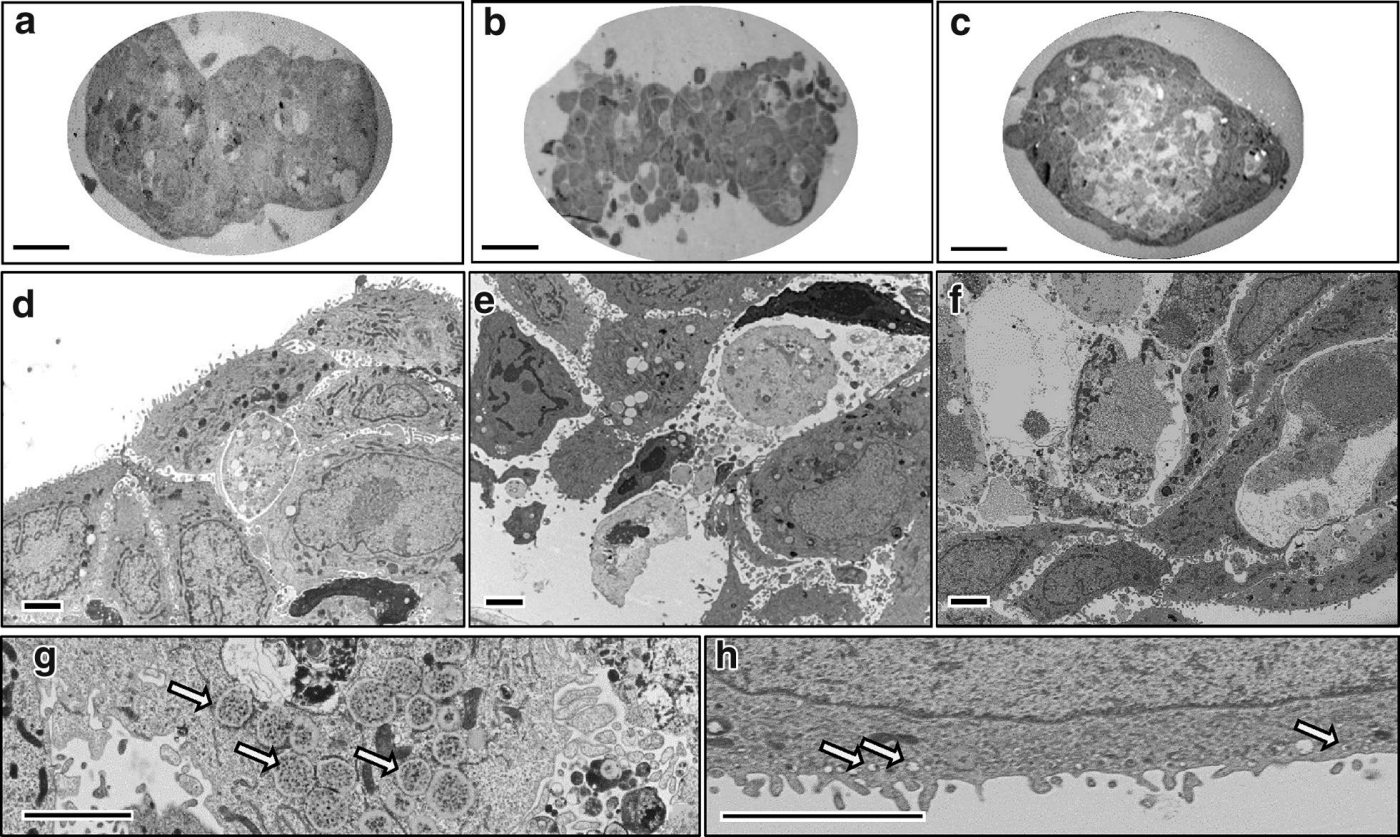
Transmission electron microscopy images of CAL-33 spheroids. Untreated control (**a**, **d**) and incubations with mTHPC (**b**, **e**, **g**) or 50 nm M-Lipidots (**c**, **f**, **h**). *Arrows* (**g**) vesicles with precipitate. *Arrows* (**h**) engulfed Lipidots. Concentration 3.67 µM mTHPC. Incubation time 24 h. Irradiation 1 min at 3440 lx. *Scale bar* (**a**–**c**) 50 µm. *Scale bar* (**d**–**h**) 2.5 µm

An investigation of CAL-33 spheroids at the ultrastructural level with electron microscopy confirmed different modes of cell death as observed after PDT with mTHPC or 50 nm M-Lipidots (Fig. 8). Untreated controls showed intact spheroid structures and most cells displayed well preserved cell organelles (Fig. 8a, d). MTHPC-induced PDT seemed to disrupt spheroid structure as a whole, causing cells to die either in an apoptotic or in a necrotic manner (Fig. 8b, e). Apoptosis was recognizable by the condensed chromatin structure and well preserved cell membranes of some dying cells. However, necrotic features like destroyed cell organelles and membranous cellular debris were present as well. Inside several cells inclusion bodies with grainy deposits were visible that may be aggregated and contrasted mTHPC (Fig. 8g). PDT with 50 nm M-Lipidots was primarily damaging the spheroid center leaving an outer rim of cells intact under these conditions (Fig. 8c). In the spheroid center cells were primarily showing features of apoptotic cell death, as described above (Fig. 8f). Additionally, in the outer cell layer, close to the cytoplasmic membrane, vesicles with enclosed sphere-like structures of about 50 nm were present that may represent M-Lipidots (Fig. 8h).

### Lipidot-PDT affects similar pathways as mTHPC-PDT

**Fig. 9:**
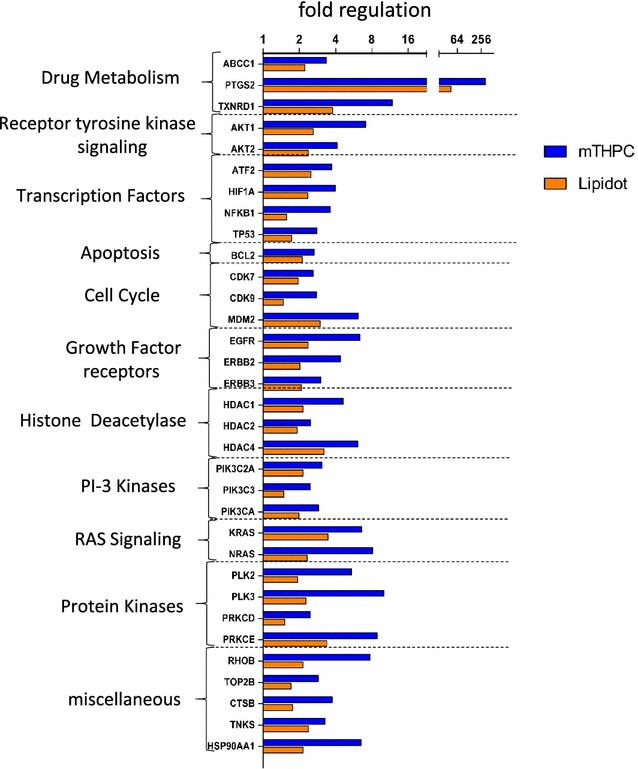
Fold expression change in spheroids after 24 h incubation with 3.67 µM mTHPC or 50 nm M-Lipidots and light illumination from 2.5 cm above with white light for 1 min at 3440 lx. Gene expression data was normalized against an untreated control and the RPLP0 house keeping gene. *CFTR/MRP* ATP-Binding Cassette, Sub-Family C, *ABCC1* Member 1, *PTGS2* Prostaglandin-Endoperoxide Synthase 2, *TXNRD1* Thioredoxin Reductase 1, *AKT1* V-Akt Murine Thymoma Viral Oncogene Homolog 1, *AKT2* V-Akt Murine Thymoma Viral Oncogene Homolog 2, *ATF2* Activating Transcription Factor 2, *HIF1A* Hypoxia Inducible Factor 1, Alpha Subunit, *NFKB1* Nuclear Factor Of Kappa Light Polypeptide Gene Enhancer In B-Cells 1, *TP53* Tumor Protein P53, *BCL2* B Cell CLL/Lymphoma 2, *CDK7* Cyclin-Dependent Kinase 7, *CDK9* Cyclin-Dependent Kinase 9, *MDM2* MDM2 Proto-Oncogene, E3, *EGFR* Epidermal Growth Factor Receptor, *ERBB2* Erb-B2 Receptor Tyrosine Kinase 2, *ERBB3* Erb-B2 Receptor Tyrosine Kinase 3, *HDAC1* Histone Deacetylase 1, *HDAC2* Histone Deacetylase 2, *HDAC4* Histone Deacetylase 4, *PIK3C2A* Phosphatidylinositol-4-Phosphate 3-Kinase, Catalytic Subunit Type 2 Alpha, *PIK3C3* Phosphatidylinositol 3-Kinase, Catalytic Subunit Type 3, *PIK3CA* Phosphatidylinositol-4,5-Bisphosphate 3-Kinase, Catalytic Subunit Alpha, *KRAS* Kirsten Rat Sarcoma Viral Oncogene Homolog, *V-Ras* Neuroblastoma RAS Viral, *NRAS* Oncogene Homolog, *PLK2* Polo-Like Kinase 2, *PLK3* Polo-Like Kinase 3, *PRKCD* Protein Kinase C, Delta, *PRKCE* Protein Kinase C, Epsilon, *RHOB* Ras Homolog Family Member B, *TOP2B* Topoisomerase (DNA) II Beta 180 kDa, *CTSB* Cathepsin B, *TNKS* Tankyrase, and Heat Shock Protein 90 kDa Alpha (Cytosolic), Class A Member 1 (HSP90AA1)

To further explore possible differences between mTHPC- and 50 nm M-Lipidot-mediated PDT, we analyzed the expression of 84 known cancer drug target genes by means of qRT-PCR (Fig. 9). Compared to the untreated control, no gross differences in overall expression patterns could be discovered after PDT, since the same 33 genes were upregulated after both regimes. However, the upregulation was generally stronger after mTHPC-PDT. This was e.g. obvious for the expression of PTGS2, TXNRD1, AKT1, NFKB1, EGFR, PIK3C3, NRAS, PLK2, PLK3, RHOB, and HSP90AA1, where a more than twofold higher upregulation was found after mTHPC-PDT compared to M-Lipidot-PDT. However, it should be noted that the same pathways were affected in the same direction (only upregulation, no downregulation) after both PDT regimes. Among others, we detected signs for abnormal regulation of KRAS and NRAS and an increase of transcription factors ATF2, HIF1A, NFKB1, TP53 despite of the upregulation of histone deacetylases HDAC1, HDAC2 and HDAC4. Genes that were not expressed and/or unaltered after both PDT regimes are summarized in Additional file 1: Table S1.

## Discussion

The powerful PS mTHPC is approved in several European countries for palliative PDT of patients with advanced head and neck cancer. However, mTHPC formulations that e.g. improve solubility of this highly hydrophobic drug, reduce its dark toxicity, enhance its intratumoral accumulation and/or increase PDT efficacy would be beneficial for systemic clinical applications [18].

Recently, we introduced solid lipid nanoparticles as stable, easy to produce and efficient carriers for mTHPC [15]. However, while physico-chemical and photophysical evaluations indicated their excellent suitability for PDT, only scarce information is available yet with regard to their behavior in biological systems. In the present study we have therefore chosen an advanced in vitro cancer spheroid model to investigate for the first time PDT effects of these particles (called M-Lipidots) at the cellular level and compare it to effects of free mTHPC. Cancer spheroids are multicellular 3D grown minitumors that display features which better mimic the biology of solid tumors than standard monolayer cultures, among others in terms of intercellular contacts, matrix deposition, physiological barriers, cellular inhomogeneity or proliferation properties [19]. Also with regard to ROS diffusion and PS penetration a 3D environment may be advantageous. Spheroids have thus been proposed not only as superior predictive platforms for testing of drugs but also of drug delivery systems [20].

Since diameters of Lipidots can be reliably adjusted between 30 and 120 nm by varying wax, oil and surfactant content [15], we have here included two exemplary sizes of mTHPC-Lipidots, namely 50 and 120 nm. In both, monolayer cultures (that served as a reference) and spheroids, we found that free mTHPC was taken up in a shorter time frame compared to mTHPC encapsulated into Lipidots. The quicker and higher accumulation of free mTHPC may be explained by the fact that lipophilic PSs can bind to serum proteins and uptake can be mediated by low lipid density protein receptors, which is considered an efficient mechanism [18]. For in vivo applications this slower accumulation of M-Lipidots must of course be considered but may be outweighed by advantages of Lipidot’s PEG chains that offer a stealth mechanism to avoid fast recognition by the immune system [21].

Our experiments further indicated favored uptake and superior spheroid penetration properties of the 50 nm M-Lipidots over the 120 nm M-Lipidots. These results are in accordance with most literature reports for other nanocomposites which suggest a size dependency of the uptake behavior and smaller diameters being more readily internalized by cells in monolayers [22]. There are fewer studies investigating size dependent penetration of nanoparticles into spheroids, however, in a work with gold nanocomposites the authors also reported a superior uptake of smaller 50 nm particles over larger 100 nm ones [23]. However, we cannot exclude that the stronger fluorescence signal observed after incubation with 50 nm M-Lipidots may also be due to the fact that five-fold more particles were present in the working solution of 50 nm M-Lipidots compared to 120 nm M-Lipidots. This is related to manufacturing processes and the aim to reach equivalent mTHPC concentrations with both M-Lipidot sizes. Furthermore, fluorescence with the nanoparticles was markedly weaker when compared to free mTHPC which is why we cannot exclude that quenching effects occur in the presence of cells.

For the following PDT experiments, we used a white light source rather than a laser to activate the PS. In a previous study we have shown that this is perfectly feasible and may be advantageous to detect (subtle) differences between effects of treatment regimes [24]. With the aim to preserve some morphology and avoid complete RNA degradation [25, 26] for our microscopic and RNA studies, we furthermore had to reduce the illumination time from 20 to 1 min. We observed a strong and comparable light-induced destruction of spheroids exposed to free mTHPC or 50 nm M-Lipidots. This similar PDT efficiency was despite our observation of a slightly different microscopic fluorescence distribution pattern within the spheroid of free mTHPC and M-Lipidots, respectively. The observed PDT effects complemented our previous study, where we have shown in a cell-free environment that 30, 50 and 100 nm mTHPC-Lipidots are capable of producing high quantum yields after illumination and that singlet oxygen may diffuse through the Lipidot shell to the surrounding [15]. As predicted because of their observed delayed and weaker cellular uptake, 120 nm M-Lipidots caused almost no PDT effects under the applied mild activation conditions. While effects may be improved with stronger illumination regimes, we have shown previously that ROS diffusion from larger Lipidots is anyway worse than from smaller ones [15].

From EM studies and the apoptosis assay, it was evident that spheroid centers were more damaged after PDT with the 50 nm Lipidots, although fluorescence accumulation was highest at the spheroid periphery. We propose that a decreasing nutrient gradient towards the spheroid center may render those cells more susceptible to PDT, and therefore also low PS doses will be sufficient to kill them.

Although PDT with both the free PS as well as the 50 nm M-Lipidots efficiently destroyed spheroids, underlying mechanisms turned out to feature differences under our experimental conditions, i.e. necrosis and apoptosis with mTHPC-PDT, and apoptosis with 50 nm M-Lipidot-PDT. The reasons for that are not clear yet. While it is well known that the subcellular localization of a PS governs PDT cell death pathways [27], we found similar cytoplasmic fluorescence patterns of mTHPC with both formulations. However, necrotic mechanisms have been reported to occur with stronger cellular photodamage [27]. Since light doses were the same, it may therefore be speculated that under the same conditions treatment with M-Lipidots initiated slightly milder PDT effects than free mTHPC. Whether this is a consequence of quantitative PS uptake, exact intracellular distribution or the nanocarrier has to be investigated.

Stronger photodamage after PDT with free mTHPC may also be concluded from our RNA expression studies where we detected always a more pronounced gene regulation. For several genes a more than twofold higher upregulation was found after mTHPC-PDT compared to M-Lipidot-PDT. As the same pathways were affected in the same direction (only upregulation, no downregulation) after both PDT regimes, it indicates common mechanisms of free and Lipidot-encapsulated mTHPC. The changed expression patterns reflect the cell’s complex acute responses to (oxidative) stress due to our PDT regimes. Many of the upregulated genes may have dual roles for apoptosis or anti-apoptosis and it is not clear yet whether we observe the cell’s efforts to initiate rescue mechanisms or the beginning of cell death. Apparently, many different pathways are dysregulated in parallel. Among others, we detected signs for abnormal regulation of the RAS signalling pathway, chromatin remodeling or an increase of transcription factor RNA despite of the upregulation of histone deacetylases.

In accordance with our previous studies with 30, 50 and 100 nm particles in MCF-7 monolayer cultures [14] the biocompatibility of empty 50 and 120 nm Lipidots could be here confirmed for CAL-33 spheroids. The observed slightly higher cytotoxicity of 120 nm Lipidots may be caused by their increased lipid concentration compared to 50 nm Lipidots, as observed before [14]. However, this difference may not be biologically relevant, leaving more than 90 % of cells vital.

Dark toxicity of PSs is an important issue for clinical PDT applications that may cause detrimental effects on healthy cells. This also applies to the strong PS mTHPC for which cytotoxic effects without light activation are well known. In our spheroid model we could demonstrate that the encapsulation of mTHPC into Lipidots significantly reduced unwanted dark toxicity of this PS at high concentrations. However, we cannot exclude that the lower toxicity is at least partially due to a lower uptake of mTHPC into the cells. Still, considering the outstanding biocompatibility of our carrier it may allow for systemic applications of higher doses of mTHPC for improved PDT without the risk for light-independent effects in patients.

Various different approaches have been proposed in the past, including the development of e.g. liposomal mTHPC formulations [28, 29] or the encapsulation of mTHPC into nanoparticles composed of poly(lactic-co-glycolic acid) [30], poly(lactic-co-glycolic acid)-b-poly(ethylene glycol) [31], poly(ethylene glycol) methacrylate-co-2-(diisopropylamino)ethyl methacrylate copolymers [32], human serum albumin [33], organic-modified silica [34] or calcium phosphate. [35] These studies describe promising carriers for mTHPC by improving solubility and reducing dark toxicity however it is not possible to directly compare them as very different model systems were used in each case. Furthermore, nanotoxicology will be very different depending on the materials used in the formulation and can differ greatly between in vitro and in vivo studies.

The 50 nm Lipidots display several favorable characteristics with regard to in vivo applications. Concerning size Tang et al. [36] e.g. could show in vivo that tumor permeation and retention of 50 nm silica particles (the EPR effect) was superior to smaller 20 nm ones and larger 200 nm ones. Furthermore, in two of our former in vivo studies with Lipidots as carrier for indocyanine green we could report on high chemical stability of the particles of over 6 months and a prolonged tumor labelling of over 1 day [7, 37]. Additionally, Lipidots displayed good long-term plasma stability and tolerability with low hemolytic activity [7, 37].

## Conclusions

In conclusion, in an advanced 3D cell culture model, 50 nm Lipidots have presented themselves as nontoxic nanocarriers for hydrophobic photosensitizers such as mTHPC that preserve its functionality in PDT. Lipidots are not only fully biocompatible and easy to produce, but may solve two important problems of mTHPC that currently prevent a more widespread clinical use of this efficient PS by rendering it water soluble and reducing its dark toxicity. The slightly milder PDT effects with M-Lipidots may be beneficial in certain clinical settings, e.g. where an apoptotic cell death (without inflammation) is clinically preferred, such as for tumor ablation.

## Additional file

10.1186/s12951-016-0221-x Additional material.

## Abbreviations

PDT: photodynamic therapy
PS: photosensitizer
FDA: (US) food and drug administration
MTHPC: m-tetrahydroxyphenylchlorin
ATP: adenosine triphosphate
EMA: European Medicines Agency
PEG: polyethylene glycol
ROS: reactive oxygen species
DiD: 1,1′-dioctadecyl-3,3,3′,3′-tetramethylindodicarbocyanine perchlorate
PBS: phosphate buffered saline
FA: formaldehyde
ROI: region of interest
CDNA: complementary DNA
FLICA: fluorochrome-labeled inhibitor of caspases
M-Lipidots: mTHPC loaded Lipidots
D-Lipidots: dye loaded Lipidots
QRT-PCR: quantitative reverse transcription polymerase chain reaction
