# Supplementary material for: Photoinduced effects of m-tetrahydroxyphenylchlorin loaded lipid nanoemulsions on multicellular tumor spheroids
Source: J Nanobiotechnology. 2016 Sep 7;14(1):68. doi: 10.1186/s12951-016-0221-x (PMC5015221; doi:10.1186/s12951-016-0221-x)
Supplement: Supplementary file 1 — 10.1186/s12951-016-0221-x Additional material. [file 12951_2016_221_MOESM1_ESM.doc]

**Supplementary Material**


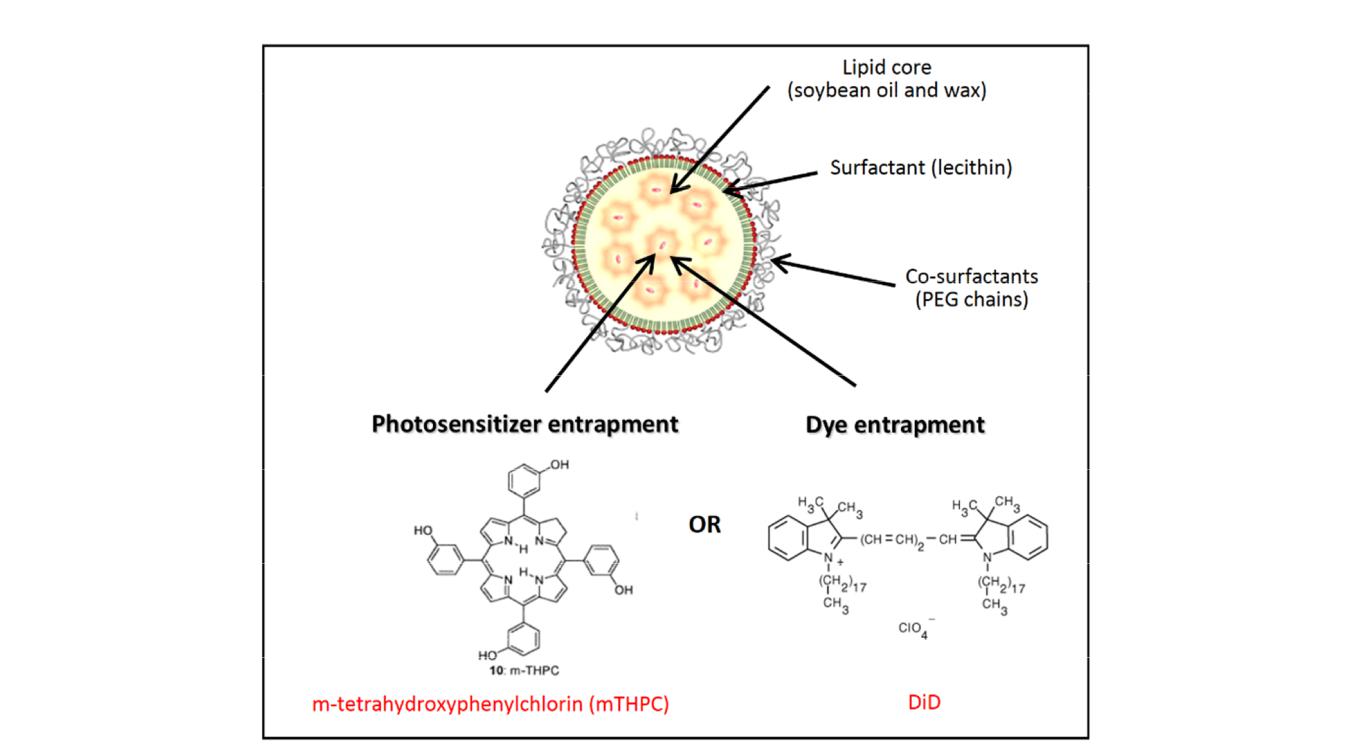


Fig. 1. Structure of Lipidot. Amphiphilic molecules like the photosensitizer mTHPC (M-Lipidot) or lipohilic dyes as DID (D-Lipidots) can be encapsulated with high efficiency.

**Electron microscopy**

Spheroids were incubated for 24 h with 3.67 µM mTHPC or 50 nm M-Lipidots and irradiated for 1 minute as described above. One h after light treatment they were washed and fixed with 2.5% glutaraldehyde in 0.1 M cacodylate buffer for 16 hours and sequentially treated with 1% OsO_4_ for 40 minutes at 0°C and 1% uranylacetate for 1 hour at room temperature.

The spheroids were dehydrated in an ethanol series and embedded in Epon/Araldite. Ultrathin (50 nm) sections were contrasted with uranyl acetate and lead citrate and were examined with a CM100 transmission electron microscope (FEI, Eindhoven, The Netherlands)

using an Orius 1000 digital camera (Gatan, Munich, Germany) or with an Auriga 40 scanning electron microscope (Zeiss, Oberkochen, Germany) using the Inlens secondary electron detector. For the investigation in the scanning electron microscope, ultrathin sections were applied on wafer and contrasted in the same way as for transmission electron microscopy.

**Quantitative reverse transcription polymerase chain reaction (qRT-PCR)**

RNA quality was assessed with the Bioanalyzer 2100 (Agilent Technologies, Basel, Switzerland). Subsequent cDNA synthesis was performed with pooled RNA from two independent experiments with the QuantiTect Reverse Transcription Kit (Qiagen) as described by manufacturer’s instructions. The obtained cDNA was further used for a quantitative PCR array (Human Cancer Drug Targets RT² Profiler PCR Array, Qiagen) performed on a LightCycler 480 (Roche, Rotkreuz, Switzerland) that allows for the investigation of 84 putative cancer drug target genes (for a list please refer to http://www.sabiosciences.com/rt_pcr_product/HTML/PAHS-507Z.html). The PCR arrays were independently repeated three times for each condition and gene expression data was analyzed with LightCycler 480 software (Roche) and SA biosciences analysis software (Qiagen).

Table S1

Quantitative PCR: list of genes whose RNA expression levels did not change 2h after PDT in CAL-33 spheroids (incubation for 24 h with 3.67 µM mTHPC or M-Lipidots, illumination for 1 min with white light (3440 lx) from 2.5 cm above). The term “expressed” refers to threshold cycle numbers (CTs) below 30; “weakly expressed” to CTs between 30 and 34.99 and “not expressed” to CTs from 35. Average results of 3 independent experiments.

|  |  |  | **Expression** |
| --- | --- | --- | --- |
| **Pathway or Family** | **Name** | **Gene** | **levels** |
|  |  |  | **before PDT** |
|  |  |  |  |
| Apoptosis | Baculoviral IAP Repeat Containing 5 | BIRC5 | weakly expressed |
|  |  |  |  |
| PI-3 Kinases & | Mechanistic Target Of Rapamycin | MTOR | expressed |
| Phosphatases |  |  |  |
|  |  |  |  |
|  |  |  |  |
|  | Erb-B2 Receptor Tyrosine Kinase 2 | ERBB4 | not expressed |
|  |  |  |  |
|  | Vascular Endothelial Growth Factor D | FIGF | weakly expressed |
|  |  |  |  |
|  | Fms-Related Tyrosine Kinase 1 | FLT1 | not expressed |
|  |  |  |  |
|  | Fms-Related Tyrosine Kinase 4 | FLT4 | not expressed |
|  |  |  |  |
|  | Insulin-Like Growth Factor 1 | IGF1 | not expressed |
|  |  |  |  |
| Growth Factors & | Insulin-Like Growth Factor 1 Receptor | IGF1R | expressed |
|  |  |  |  |
|  | Insulin-Like Growth Factor 1 | IGF2 | not expressed |
| Receptors |  |  |  |
|  |  |  |  |
|  | Kinase Insert Domain Receptor | KDR | not expressed |
|  |  |  |  |
|  | V-Kit Hardy-Zuckerman 4 Feline | KIT | weakly expressed |
|  | Sarcoma Viral Oncogene Homolog |  |  |
|  |  |  |  |
|  |  |  |  |
|  | Platelet-Derived Growth Factor | PDGFRA | not expressed |
|  | Receptor, Alpha Polypeptide |  |  |
|  |  |  |  |
|  |  |  |  |
|  | Platelet-Derived Growth Factor | PDGFRB | not expressed |
|  | Receptor, Beta Polypeptide |  |  |
|  |  |  |  |
|  |  |  |  |
| Drug Metabolism | Glutathione S-Transferase Pi 1 | GSTP1 | expressed |
|  |  |  |  |
|  | Thioredoxin | TXN | expressed |
|  |  |  |  |
|  |  |  |  |
| G Protein Signaling | Ras Homolog Family Member A | RHOA | expressed |
|  |  |  |  |
|  | Estrogen Receptor 1 | ESR1 | weakly expressed |
|  |  |  |  |
| Hormone Receptors | Estrogen Receptor 2 | ESR2 | not expressed |
|  |  |  |  |
|  | Progesterone Receptor | PGR | not expressed |
|  |  |  |  |
| Heat Shock Proteins | Heat Shock Protein 90kDa Beta | HSP90B1 | expressed |
|  | (Grp94), Member 1 |  |  |
|  |  |  |  |
|  |  |  |  |
|  |  |  |  |

| Receptor Tyrosine | Growth Factor Receptor-Bound | GRB2 | expressed |
| --- | --- | --- | --- |
| Kinase Signaling | Protein 2 |  |  |
|  |  |  |  |
|  |  |  |  |
|  | Cathepsin D | CTSD | expressed |
|  |  |  |  |
| Cathepsins | Cathepsin L | CTSL1 | expressed |
|  |  |  |  |
|  | Cathepsin S | CTSS | expressed |
|  |  |  |  |
|  | Cyclin-Dependent Kinase 1 | CDK1 | expressed |
|  |  |  |  |
|  | Cell Division Cycle 25A | CDC25A | weakly expressed |
|  |  |  |  |
|  | Cyclin-Dependent Kinase 2 | CDK2 | weakly expressed |
|  |  |  |  |
| Cell Cycle | Cyclin-Dependent Kinase 4 | CDK4 | expressed |
|  |  |  |  |
|  | Cyclin-Dependent Kinase 5 | CDK5 | expressed |
|  |  |  |  |
|  | Cyclin-Dependent Kinase 8 | CDK8 | weakly expressed |
|  |  |  |  |
|  | MDM4, P53 Regulator | MDM4 | expressed |
|  |  |  |  |
| Topoisomerases, Type II | Telomerase Reverse Transcriptase | TERT | not expressed |
|  |  |  |  |
|  | Topoisomerase (DNA) II Alpha 170kDa | TOP2A | weakly expressed |
|  |  |  |  |
|  |  |  |  |
| Transcription Factors | Interferon Regulatory Factor | IRF5 | weakly expressed |
|  |  |  |  |
|  | Aurora Kinase A | AURKA | expressed |
|  |  |  |  |
|  | Aurora Kinase B | AURKB | weakly expressed |
|  |  |  |  |
| Protein Kinases | Aurora Kinase C | AURKC | weakly expressed |
|  |  |  |  |
|  | Polo-Like Kinase 1 | PLK1 | expressed |
|  |  |  |  |
|  |  |  |  |
|  | Polo-Like Kinase 4 | PLK4 | weakly expressed |
|  |  |  |  |
|  | Protein Interacting With PRKCA 1 | PRKCA | weakly expressed |
|  |  |  |  |
| RAS Signaling | Harvey Rat Sarcoma Viral Oncogene | HRAS | expressed |
|  | Homolog |  |  |
|  |  |  |  |
|  |  |  |  |
|  | Histone Deacetylase 11 | HDAC11 | expressed |
|  |  |  |  |
|  | Histone Deacetylase 13 | HDAC3 | expressed |
|  |  |  |  |
| Histone Deacetylases | Histone Deacetylase 6 | HDAC6 | weakly expressed |
|  |  |  |  |
|  | Histone Deacetylase 17 | HDAC7 | expressed |
|  |  |  |  |
|  | Histone Deacetylase 8 | HDAC8 | weakly expressed |
|  |  |  |  |
|  | Poly (ADP-Ribose) Polymerase 1 | PARP1 | weakly expressed |
| Poly ADP-Ribose |  |  |  |
|  | Poly (ADP-Ribose) Polymerase 2 | PARP2 | weakly expressed |
| Polymerases |  |  |  |
|  |  |  |  |
|  | Poly (ADP-Ribose) Polymerase 4 | PARP4 | expressed |
|  |  |  |  |
|  |  |  |  |
| Structural Protein | Netrin 3 | NTN3 | weakly expressed |
|  |  |  |  |
